# Does Self-Rated Health Foretell Loss of Dual Functionality?

**DOI:** 10.1093/geroni/igaf122.085

**Published:** 2025-12-31

**Authors:** Kenneth Ferraro, Mallory Bell

**Affiliations:** Purdue University, West Lafayette, Indiana, United States; Purdue, Lafayette, Indiana, United States

## Abstract

Self-rated health (SRH) has been described as a global measure of health because the respondent is asked to reply based on one’s assessment of overall health. Although there is a rich literature examining whether SRH predicts a variety of single measures of health, it is unknown whether it also predicts a global measure of health that is defined by a combination of physical and cognitive functions. Given that most people aspire to retain their physical function and cognitive function, we investigate if SRH is a valid predictor of dual functionality (DF). We use data from the Health and Retirement Study from 2004 to 2020 to prospectively study the relationship between SRH and DF. We used Weibull accelerated failure-time (WAFT) models with age as the time metric to investigate whether SRH is associated with the loss of DF. Among respondents with DF at baseline, each higher level of SRH was significantly related to DF in fully-adjusted models. Using poor SRH as the reference group, even respondents in fair health reported a 10% later loss of DF (time ratio = 1.10, SE = .02, p < .001). Compared to poor SRH, respondents who rated their health as good reported a 19% later loss of DF; those who rated their health as very good reported a 28% later loss of DF; and respondents who rated their health as excellent reported an impressive 35% later loss of DF (p < .001). We conclude that SRH is a valuable upstream predictor of DF.

